# General practitioners’ involvement in oncological aftercare (ACTION): a study protocol for a mixed-methods study

**DOI:** 10.1186/s12875-026-03603-z

**Published:** 2026-10-13

**Authors:** Katharina van Baal, Stephanie Stiel, Svenja Arendt, Catharina Münte, Malte Klemmt

**Affiliations:** Institute for General Practice and Palliative Care, Hannover Medical School, Carl-Neuberg-Straße 1, 30625 Hannover, Germany

**Keywords:** Cancer, Aftercare, General practitioner, Health services research, Study protocol

## Abstract

**Background:**

The growing number of cancer survivors in Germany necessitates sustainable, interdisciplinary aftercare. General practitioners (GPs) are well-positioned to provide long-term, trust-based support, including early detection of recurrences and late effects. However, evidence on the level of GP involvement in oncological aftercare, on interdisciplinary aftercare models involving GPs and specialists, and on associated barriers and facilitators remains limited.

**Methods:**

The project “general practitioners’ involvement in oncological aftercare” (ACTION; funded by the German Cancer Aid; grant number 70116960) will employ a mixed-methods approach across two phases. In phase 1, experiences with GPs involvement in oncological aftercare will be examined. Therefore, routine data from the AOK Lower Saxony and the Lower Saxony Cancer Registry will be analysed retrospectively (WP1a), supplemented by surveys of GPs (WP1b) and oncologists (WP1c), and qualitative interviews with patients (WP1d). Phase 2 will then build on the results from phase 1 to develop and agree on recommendations for GP involvement in oncological aftercare. An expert workshop will aim to develop structured recommendations for enhanced GP involvement (WP2a), which are refined via a Delphi consensus process (WP2b).

**Discussion:**

The ACTION project will generate new insights into the involvement of GPs in oncological aftercare and contribute to adequately addressing future challenges in patient care. It will stimulate the empirically supported development and implementation of interdisciplinary aftercare models in clinical practice and has thus the chance to improve the long-term care of cancer survivors in Germany.

**Trial registration:**

The study was registered with the German Clinical Trials Register (Deutsches Register Klinischer Studien; DRKS00040034; date of registration: 11 May 2026).

## Background

### Oncological aftercare

Demographic change and advances in the diagnosis and treatment of cancer imply a steady increase in the number of (long-term) survivors after and with cancer [1, 2]. Aftercare for these patients is essential to detect recurrences or metastases at an early stage, to identify and treat side effects and long-term and late effects of tumor diseases and therapies, and to offer psychosocial support [2, 3]. In Germany, continuous and evidence-based aftercare is recommended by professional societies and associations [4–6] and is part of various S3 guidelines [7–10]. For example, the S3 guideline for breast cancer recommends aftercare consisting of medical history, physical examination, medical consultation, care and support, and imaging diagnostics to detect local and locoregional recurrence and contralateral breast cancer [8]. The S3 guideline for pancreatic cancer recommends regular medical aftercare, which can be provided by surgeons, internists, or attending general practitioners (GPs) [9].

Aftercare passports (“Nachsorgepässe”) can serve as a tool for information exchange among the various professions involved in oncological aftercare, aiming to keep all participating healthcare professionals up to date on the latest diagnostic and therapeutic developments through comprehensive documentation. To date, there are no uniform regulations in Germany governing the issuance of aftercare passports to cancer patients [3]. Outpatient aftercare usually begins after inpatient or outpatient therapy or medical rehabilitation [5]. The start and duration of aftercare is individual to each patient and varies in some cases depending on the tumor entity and the course of the disease or therapy and any side effects [11, 12]. There are differing opinions on whether and how tasks in outpatient oncological aftercare can be distributed effectively among the parties involved [13–16]. Those affected often do not know who the right contact persons are for individual issues in the context of aftercare [3].

### The general practitioners’ role

GPs can play an important role in the prevention, early detection, and long-term care of patients with cancer [17, 18], especially since they receive all important findings and treatment recommendations [3] and can provide low-threshold treatment [19]. The relationship between patients and their GPs has often grown over decades, resulting in a relationship of trust. In addition, GPs are usually located close to where their patients live [19]. International studies report advantages of GP-led survivorship care programs, e.g., with regard to the reduction of physical illness and treatment consequences [20, 21]. However, due to their heavy workload, not all GPs want to take on (substantially more) responsibility in this area, and there are reservations regarding resources, knowledge, and communication with the specialists involved [22–24]. At the same time, studies report that many GPs would like to participate in oncological aftercare in cooperation with oncological specialists [24, 25].

### Patients’ perspective

Most patients would like to see their GPs involved in oncological aftercare, with close cooperation between GPs and oncological specialists [26], but do not generally consider this to be part of the usual scope of general practice [27, 28]. In the case of aftercare models involving GPs and oncological specialists, patients believe that evidence is needed regarding the effectiveness of these models, freedom of choice, addressing concerns about the capacities of GPs, specialized oncological knowledge, and clear communication between all parties involved [26, 29].

### International findings

Aftercare models in which GPs are actively involved and express confidence in their competence in oncological aftercare already exist [19, 22, 30]. Both GPs and oncological specialists report acceptance of joint follow-up care models and consider early and continuous cooperation in the care processes to be particularly important [26]. A cross-sectional clinical study shows a high degree of agreement between GPs and oncological specialists in the evaluation of patient aftercare [31]. Although barriers to its implementation in healthcare practice remain, there is clear evidence that greater involvement of GPs in oncological aftercare can improve patient care. Systematic reviews report an improvement in the physical and psychosocial well-being of patients, a higher subjective perception of continuity of treatment, and less travel for patients [32, 33]. Study findings also suggest that GP-led aftercare is equivalent to specialist care in terms of patient well-being, recurrence rates, and survival rates, and may be less costly [34, 35]. In this context, Schütze et al. note that greater involvement of GPs in aftercare could reduce the burden on specialist oncological clinics [26]. A randomized controlled trial investigating the provision of more information towards GPs by oncological specialists through an expanded information routine found that GPs gained more knowledge and understanding of the disease and their patients’ treatments, enabling them to better identify and respond to patients’ support needs [36].

### Research gap

Due to the continuing increase in the number of cancer patients in Germany [37], new aftercare models involving various stakeholders in outpatient and inpatient care are needed, e.g., with close, early involvement of GPs. It is currently unclear for the German healthcare sector whether and how interdisciplinary aftercare models involving GPs and oncological specialists are being implemented. Specifically, it remains unclear to what extent GPs are actually involved and to what extent they would like to be involved in oncological aftercare in the future. There is also a lack of data on the challenges with GP participation in oncological aftercare from the perspective of the various parties involved. Furthermore, it is not known whether and how GP participation in oncological aftercare needs to be optimised, and whether the aftercare passport can be a suitable tool for improving interdisciplinary cooperation in oncological aftercare in everyday healthcare.

### Aims

Based on these gaps in the empirical evidence, the overarching aim of the project “general practitioners’ involvement in oncological aftercare (ACTION)” is to explore experiences and perspectives on GP involvement in oncological aftercare from multiple perspectives and to derive and agree on recommendations for optimising GP involvement in oncological aftercare.

The specific aims are:

1. To examine the use and remuneration of oncological aftercare services and their compliance with recommended aftercare regimens, as well as the caregivers involved, using three tumor entities as examples.
2. To describe the experiences of GPs with participation in oncological aftercare, as well as barriers and facilitating factors for closer involvement of GPs.
3. To explore the experiences of oncological specialists with GP involvement in oncological aftercare, as well as specific expectations and requirements for GP involvement.
4. To explore the experiences of patients with GP involvement in oncological aftercare, as well as specific expectations and requirements for GP involvement.
5. To evaluate the use and practical benefits of aftercare passports from the perspective of GPs, oncological specialists, and patients.
6. To develop and agree on recommendations for an optimised involvement of GPs in oncological aftercare and an increased implementation of interdisciplinary aftercare models.

The long-term goal is to enable the empirically supported development of interdisciplinary aftercare models with the participation of GPs and their implementation in everyday care. This contributes to the needs based and future-oriented optimisation of oncological aftercare. In the long term, this can improve the care of cancer survivors in oncological aftercare.

### Research questions

1. Which medical groups provide (what) oncological aftercare services or bill for them, and to what extent does patients’ use of oncological aftercare correspond to the recommended aftercare regimens (using the example of three tumor entities)?
2. What experiences do GPs have with participating in oncological aftercare, and what barriers and facilitators are relevant to them for closer involvement in oncological aftercare?
3. What experiences do oncological specialists have with GP involvement in oncological aftercare, and what specific expectations and requirements do they have for GP involvement?
4. What experiences do patients have with GP involvement in oncological aftercare, and what specific expectations and requirements do they have for GP involvement?
5. How and for whom are aftercare passports used, and how do GPs, oncological specialists, and patients assess their practical usefulness?
6. What recommendations for closer involvement of GPs in oncological aftercare and for the development and implementation of interdisciplinary aftercare models can be empirically derived and agreed upon?

## Methods/Design

### Study design

The ACTION project (funded by the German Cancer Aid; grant number 70116960; funding period November 2026 - April 2029) is a mixed-method, non-interventional, cross-sectional study using qualitative and quantitative methodological approaches.

### Study phases

**Fig. 1:**
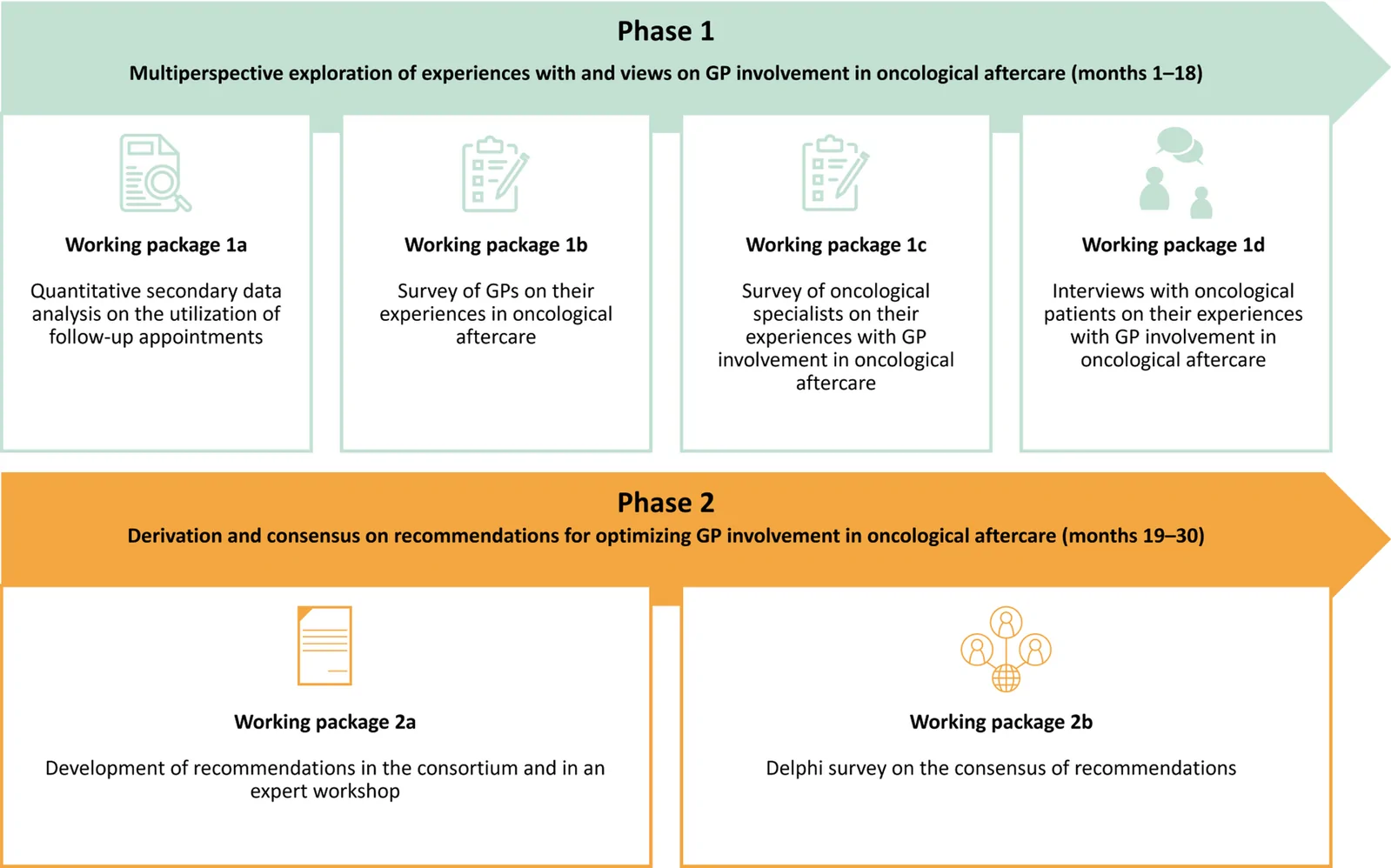
Work program in the ACTION project

The ACTION project with a duration of 30 months comprises two consecutive project phases with six working packages (WP) in which mixed methods are used to answer the research questions (Fig. 1). Recruitment of the first study participants is expected to begin in the spring of 2027. Data collection is expected to be completed in 2029. Initial results will be available depending on the completion of the work packages, but no earlier than the fall of 2027.

### Phase 1: multiperspective exploration of experiences with and views on GP involvement in oncological aftercare

#### Setting

In phase 1, the focus is on surveying the experiences and perspectives of various stakeholders on oncological aftercare in the federal state of Lower Saxony. Lower Saxony is a large state and, like many other regions in Germany, it has a mixed urban-rural population and infrastructure and is therefore comparable to other states and regions within Germany.

#### Target groups

In phase 1, three target groups are addressed:

1) GPs: specialists in general medicine, or in internal medicine working in primary care in Lower Saxony.

2) Oncological specialists: Practicing oncologists (specialists in internal medicine, haematology, oncology) as well as oncologists working in oncology centres, organ cancer centres, and oncology outpatient clinics in Lower Saxony.

3) Oncological patients: Patients with oncological diseases who have completed primary therapy and reside in Lower Saxony.

### Work package 1a: quantitative secondary data analysis on the utilization of follow-up appointments

In WP1a, secondary data from the Lower Saxony Clinical Cancer Registry (KKN) and the Allgemeine Ortskrankenkasse Lower Saxony (AOKN) (statutory health insurance) will be analyzed with regard to the utilization of aftercare appointments after completion of primary therapy and the medical groups involved in aftercare, using three tumor entities: breast cancer, colorectal cancer (stages II and III) and melanoma. In addition, the extent to which the use of aftercare appointments corresponds to the recommended schedules in the S3 guidelines for these three selected entities is being examined.

#### Recruitment and number of cases

With more than 3 million insured persons (approx. 37% of the total population of the federal state), AOKN is the largest statutory health insurance company in Lower Saxony [38]. Since July 1, 2018, all physicians working in oncology in Lower Saxony are required to report to the KKN. This reporting requirement includes data on all treatment steps, including aftercare, and on the individual disease progression of patients with oncological diseases. The KKN makes this data available for research purposes [39]. In WP1a, routine quantitative data from the KKN is used to identify individuals with statutory health insurance from Lower Saxony who are at least 18 years old at the time of diagnosis, reside in Lower Saxony, and are insured with the AOKN. Diagnoses according to International Statistical Classification of Diseases and Related Health Problems, 10th Revision, German Modification (ICD-10-GM) for the tumor entities breast cancer (C50), colorectal cancer (C18, C19, C20), and melanoma (C43) are included. These three entities are selected because the respective S3 guidelines define specific aftercare regimens, by which the research questions in the ACTION project can be answered, and because GPs involvement seems to be reasonable due to the primarily outpatient care in many cases. This KKN data set is supplemented by data from the AOKN via a data linkage. A further inclusion criterion is then continuous health insurance with the AOKN for a period of at least one year after diagnosis. Ordinary aftercare has only been reportable to the KKN since January 1, 2021, which means that a complete follow-up is only expected from this date onwards. The aim is to include patients with corresponding diagnoses from 2021 to 2024, which would result in a follow-up period of at least 2 and at most 5 years until 2026. The number of cases is to be determined in the project. The health insurance number as a unique identifying variable enables a direct link between the KKN and AOKN data. This approach makes it possible to close data gaps in both data sets. For example, follow-up examinations are currently underrepresented in the KKN data, but information on the stage and classification of tumor diseases is included in the KKN data. The secondary data from the AOKN contains largely complete information at the individual level, but does not include specific information about diseases, such as tumor stage. By linking the two data sets, a more comprehensive picture of oncological aftercare will be obtained.

#### Data collection and analysis

The KKN and AOKN network has practical experience in performing data linkage, which will be used for consulting purposes. Health data in the sense of sensitive data in disease registries such as the KKN, as well as social data such as the AOKN’s data, are specially protected by legislation under the European General Data Protection Regulation. The project is closely monitored by the KKN’s trust area, which in turn cooperates in this matter with the responsible ministry in Lower Saxony. The details of the record linkage procedure to be used are based on the findings and descriptions of Pigeot et al. [40] and Good Practice Data Linkage [41]. The unique personal identification variable is available from the KKN and the AOKN. This allows direct data linkage and the two data sources to be linked deterministically. Specifically, the KKN selects patients who meet the inclusion criteria. The individual health insurance number of these patients is extracted and the KKN assigns a pseudonym. For this purpose, the identification variable is randomly ordered by sequential numbers are assigned an internal project identification number (study ID). The assignment is documented in the KKN’s trust domain. The KKN separately creates a pseudonymized data set with the study ID and the other variables to be analyzed from the KKN’s care data (including demographic data, diagnosis data, tumor stage/classification, reported care data including follow-up examinations, and participating medical groups). This pseudonymized KKN data set is transferred to the project team at Hannover Medical School (MHH) as the evaluating body. This prevents any conclusions being drawn from the study ID about the actual identity of individuals. The AOKN receives a file from the KKN containing two variables: the health insurance number of the selected patients and the assigned study ID. Since the AOKN already has the health insurance number for billing purposes for all its insured persons in the respective routine data and there is a reporting obligation to the KKN, the transferred file does not contain any unknown sensitive data. The AOKN then uses this study ID to create its own pseudonymized data set for the insured persons included, which contains the variables to be analysed from the AOKN’s billing data (including demographic data, diagnosis data, outpatient and inpatient services, billing medical groups). This pseudonymized AOKN data set is transferred to the project team at MHH as the evaluating body, and it is still not possible to draw conclusions about personal information. At the MHH, the transmitted pseudonymized data sets from KKN and AOKN can be merged using the case-based assigned study ID without further data protection challenges and without the involvement of another party. The procedure described was developed and agreed upon jointly with the partners KKN and AOKN during the preparations for the project application. The secondary data are analysed descriptively. To investigate potential correlations between patient characteristics and aftercare behaviour, bivariate analyses are performed using Pearson’s correlation coefficient (r) as a measure. The descriptive and bivariate analyses are performed using SPSS.

### Work package 1b: survey of GPs on their experiences in oncological aftercare

In WP1b, GPs in Lower Saxony are surveyed to gather information about their experiences and assessments of their own involvement in oncological aftercare, barriers and promoting factors for closer integration of their own discipline, and the use of aftercare passports.

#### Recruitment and number of cases

In WP1, all GPs working in Lower Saxony (~ *N* = 5,223) are addressed. The contact details of the GPs are provided by the cooperation partner AOKN and updated and completed by the project team with the help of the Association of Statutory Health Insurance Physicians of Lower Saxony (KVN). GPs will be contacted primarily by email and fax.

Inclusion criteria for participation in the survey are primarily working as a GP and the general practice to be located in Lower Saxony. GPs who exclusively treat private patients are excluded. Based on experiences from previous surveys of GPs in Lower Saxony, a response rate of approximately 10% (*n* = 522) is expected, with deviations of 10% in either direction possible (*n* = 470 to *n* = 574).

To increase the response rate, several reminders to participate in the survey will be sent via various channels. In addition, the survey is distributed via the teaching and research practice network and the social media channels of the participating institutions. Existing contacts and networks are also used to increase the response rate. These include, for example, a publication with a call for participation in the Lower Saxony Medical Journal and a placement in the newsletter of the General Practitioners’ Association Lower Saxony (HÄVN).

#### Data collection and analysis

A structured questionnaire for GPs being developed in collaboration with specialists in the field and based on current literature and previous scientific findings. Common principles of test construction, such as determining the construct to be measured and its dimensions, and defining item and response modalities, are considered. Attention will be paid to avoiding response tendencies toward the middle of scales or socially desirable responses. Items and response scales will be generated, and the questionnaire will be programmed online in SoSciSurvey [42]. The questionnaire focuses in particular on the experiences of GPs with oncological aftercare. It also aims to capture their self-image and their own role in oncological aftercare, the extent and nature of their involvement in oncological aftercare to date, and barriers and promoting factors for closer involvement in oncological aftercare. In addition, the survey addresses ways of exchanging information with oncological specialists and, overall, interprofessional communication in the context of oncological aftercare, as well as the use of aftercare passports. The questionnaire developed will be tested in a pretest with *n* = 6–8 GPs and adjusted, if necessary. The data transmitted from the online survey will be collected anonymously and output by SoSciSurvey in Excel and SPSS. Analyses will be carried out using descriptive statistics.

### Work package 1c: survey of oncological specialists on their experiences with GP involvement in oncological aftercare

In WP1c, oncological specialists are surveyed online about their experiences with GP involvement in oncological aftercare, their expectations and requirements of GPs, and their use of aftercare passports.

#### Recruitment and number of cases

WP1c addresses oncological specialists (registered oncologists as specialists in internal medicine, hematology, oncology, and employees in oncology centers, organ cancer centers, or oncology outpatient clinics). In August 2024, 270 specialists in internal medicine, hematology, and oncology were registered in the Medical Association of Lower Saxony (ÄKN). The German Cancer Society (DKG) Oncomap lists a total of 160 centers in Lower Saxony. Assuming that one person in a managerial position should participate per center, a total of approximately 430 oncological specialists in Lower Saxony will be addressed and contacted primarily by email and fax. During the survey period, several reminders to participate in the survey will be sent out via various channels in order to increase the response rate. In addition, the survey will be advertised through other channels, e.g., with the help of the German Society for Hematology and Medical Oncology (DGHO), the Association of Practicing Internal Oncologists in Lower Saxony (AIO), and the Comprehensive Cancer Center Lower Saxony (CCC-N). Oncological specialists working in the federal state of Lower Saxony will be included. Specialists who exclusively treat private patients will be excluded. Based on the described *N* = 430 oncological specialists in Lower Saxony and previous experience with the willingness of this population to participate, a response rate of 15% is anticipated. A sample size of approximately *n* = 65 oncological specialists is therefore assumed, with a possible deviation of 10% in either direction (*n* = 58 to *n* = 72).

#### Data collection and analysis

Together with the project partners and in consultation with those responsible at CCC-N and based on the literature and previous scientific findings, a questionnaire is being developed to record the experiences of oncological specialists with the involvement of GPs in oncological aftercare. Analogous to the GP questionnaire, the principles mentioned above for test construction are being applied in the development of the structured questionnaire, which is being programmed in SoSciSurvey. The survey addresses, among other things, the specialists’ self-image and their own role in oncological aftercare. It also considers expectations and requirements for GPs in their current or future involvement in oncological aftercare. Furthermore, it will focus on ways of exchanging information with other parties involved in care, including GPs, as well as interprofessional communication and the use of aftercare passports in everyday practical care.

The questionnaire developed will be tested in a pretest with *n* = 4–6 oncological specialists (access via the project’s cooperation partners) and adjusted if necessary. Using a method comparable to WP1b, the data transmitted from the online survey will be collected anonymously and exported from SoSciSurvey to Excel and SPSS. Descriptive statistics and frequency analyses will be performed.

### Work package 1d: interviews with oncological patients on their experiences with GP involvement in oncological aftercare

In WP1d, oncological patients in the aftercare stage are asked about their experiences and wishes regarding the involvement of their GPs in oncological aftercare. The interviews also focus on expectations and requirements with regard to the involvement of GPs in oncological aftercare and the personal use of aftercare passports.

#### Recruitment and number of cases

Oncological patients are recruited according to the principle of selective sampling. This ensures that the sample is heterogeneous and meets the participant characteristics relevant to the research question [43]. Contact with patients is established primarily through general practices in the teaching and research practice network of the Institute of General Medicine and Palliative Medicine (approx. 260 practices) and the oncology outpatient clinic at MHH. In addition, oncological practices, self-help groups, and patient representatives from CCC-N are involved in the recruitment. Inclusion criteria are an oncological disease and completed primary therapy, i.e., the aftercare stage. In addition, patients must have their primary residence in Lower Saxony, have been at least 18 years old at the time of diagnosis, and have sufficient German language skills to conduct an interview. The aim is to achieve the greatest possible heterogeneity regarding the characteristics like age, gender or geographic distribution. It is assumed that characteristic heterogeneity will be achieved in *n* = 25–30 interviews with patients.

#### Data collection and analysis

Guided problem-centered interviews [44] are conducted with oncological patients. The individual interviews will be conducted via telephone or video chat, depending on the patient’s preference. The interview guide is based on literature and developed in collaboration with the project partners and with the involvement of patient representatives from the CCC-N. Before use, the guide is tested with 2–3 patients or patient representatives. The interviews will focus on determining the views of those affected on the involvement of GPs in oncological aftercare. Experiences and wishes regarding the involvement of GPs in oncological aftercare will be addressed, as well as expectations and requirements with regard to the involvement of GPs in oncological aftercare. Furthermore, the use of aftercare passports will be discussed. The interview guide contains guiding questions with exploratory prompts as well as optional detailed questions to maintain the flow of the narrative and to be able to delve deeper into certain topics, if necessary. Sociodemographic data of the interview partners is collected using a short questionnaire, that is completed together with the patients after the interviews. With the consent of the interviewees, the interviews are digitally audio-recorded and transcribed anonymously. The transcripts are evaluated using content-structuring qualitative content analysis [45] with the help of MAXQDA. The sociodemographic data from the questionnaire is entered into SPSS and evaluated descriptively.

### Phase 2: derivation and consensus on recommendations for optimizing GP involvement in oncological aftercare

#### Setting

In phase 2, the results from phase 1 will be combined, and recommendations will be developed and agreed upon that are intended to apply to the whole of Germany and can bring added value to the aftercare of oncological patients.

#### Target groups

Phase 2 addresses the target group of experts in oncological care in Germany. The group of experts consists of healthcare providers from the outpatient and inpatient sectors as well as representatives of professional societies and associations. Other actors at the macro and meso levels of the healthcare system, as well as patient representatives and representatives of self-help groups, complete the group of experts.

### Work package 2a: development of recommendations in the consortium and in an expert workshop

In WP2a, recommendations for closer involvement of GPs in oncological aftercare and the implementation of joint aftercare models will be developed within the consortium and in a subsequent expert workshop. The results from phase 1 (WP1a-1d) will first be compiled and reviewed within the consortium in order to prepare for the expert workshop. From this material, the project team will derive initial recommendations for closer involvement of GPs in oncological aftercare and for the implementation of joint aftercare models. These recommendations will form the basis for the expert workshop.

#### Recruitment and number of cases

Participants in the expert workshop are a diverse group of healthcare providers (GPs and oncological specialists from different settings), representatives of professional societies and associations and other actors at the macro and meso levels (e.g., representatives of the German Cancer Research Center (DKFZ), regional cancer counseling centers, patient representatives, participants in the Oncology Guidelines Program). There may be some overlap with participants from phase 1. A total of *n* = 20 experts from across Germany are expected to participate in the workshop. Based on previous project experience, approximately 35 individuals with expertise in primary care and/or oncological care with a focus on aftercare care will be contacted in advance and informed about the project to reach a total of 20 experts willing to participate. An expense allowance and reimbursement of travel expenses is offered for participation in the workshop.

#### Data collection and analysis

The expert workshop is to take place on site in Hanover in order to ensure the best possible exchange between all participants. The results developed in advance by the consortium will be presented and discussed in an approximately 4-hour workshop with experts. The exchange and discussion with the experts aim to further develop the results obtained so far and to identify additional factors that promote or hinder the involvement of GPs in oncological aftercare and the implementation of joint aftercare models. The focus is on the evidence-based development of recommendations for closer involvement of GPs in oncological aftercare and the collection of ideas for joint aftercare models and their implementation. Methodologically, the implementation workshop is based on focus group techniques [46].

After the project team has presented the results from phase 1, the participants will likely be divided into small groups of each five people, in which they will focus on a specific topic area according to their expertise. The content of the topic areas will be determined during the course of the project based on the results of WP1a-1d. The specific task for the small groups could be, for example: “Please develop specific recommendations on how to implement the involvement of general practitioners in oncological aftercare!” The results of the workshop will be translated into practice-oriented recommendations by the consortium. In the long term, the ideas collected should contribute to the needs-based and future-oriented optimization of oncological aftercare.

The sociodemographic data of the participating experts will be collected using a questionnaire. The workshop will be digitally recorded and then transcribed verbatim. Based on the transcript, a summary of key discussion points will be prepared according to Ruddat [47]. The sociodemographic data of the participants will be evaluated using descriptive statistics and frequency analyses in SPSS. The qualitative data will be evaluated using the data analysis software MAXQDA. In the follow-up to the expert workshop, the recommendations will be finalized by the project consortium and made available for WP2b.

### Work package 2b: delphi survey on the consensus of recommendations

In WP2b, recommendations for closer involvement of GPs in oncological aftercare and implementation of joint follow-up care models agreed upon in a Delphi process with experts.

#### Recruitment and number of cases

Approximately *n* = 35 experts from across Germany are to participate in the Delphi survey. Participants from the workshop (WP2a) and other relevant experts from the above-mentioned target groups will be invited. Based on previous project experience, approximately 50 people with expertise in primary care and/or oncological care with a focus on aftercare will be invited to participate in round 1. An expense allowance will be requested for participation in all two to three Delphi survey rounds.

#### Data collection and analysis

The recommendations developed by the consortium and in the expert workshop (WP2a) form the basis for the Delphi method in WP2b. The aim of the two- to three-stage Delphi survey is to further develop the recommendations for closer involvement of GPs in oncological aftercare and the implementation of joint aftercare models. The Delphi process culminates in the consensus on these recommendations, which can serve as a guideline for the optimization of oncological aftercare throughout Germany. The recommendations from WP2a will be sent to the group of experts in their final form, prepared and finalized by the project team, via a link to the SoSciSurvey survey tool. Respondents will rate each recommendation on a 4-point scale (“strongly agree” – ‘somewhat agree’ – ‘somewhat disagree’ – ‘disagree’) to indicate the extent to which, in their personal opinion, these recommendations are (a) relevant and (b) feasible/practicable in healthcare practice for closer involvement of GPs in oncological aftercare and the implementation of joint aftercare models. In particular, participants have the opportunity to critically review and comment on the jointly developed recommendations and to submit suggestions for wording. A group consensus on all recommendations is to be reached in two to three rounds of voting. A group consensus is reached when 75% of respondents (tend to) agree with a recommendation [48]. If individual recommendations do not (yet) reach a consensus in the first or second round of voting, the corresponding comments from participants in the free text fields of the online survey will be used to adjust the content and/or wording of the recommendation. All recommendations that have not (yet) been agreed upon are then presented to all participants in the previous Delphi round in the second or third Delphi round and voted on again. If no consensus is reached on individual recommendations in up to three rounds of questioning, these are not included in the final set of recommendations. The quantitative data generated from the Delphi process will be evaluated using descriptive statistics and frequency analyses in SPSS. The results, in particular non-consensus recommendations, will then be discussed by the project team. Further minor changes may be made to the recommendations if necessary.

#### Data synthesis

Data synthesis will take place at two points in the project. Firstly, after completion of the empirical surveys and data analyses and in the sense of a triangulation of methodological approaches, data, and perspectives at the end of phase 1 [49]. Secondly, following phase 2, the results from phases 1 and 2 will be combined by the consortium.

#### Ethics and data protection

The study received ethical approval from the Ethics Committee of the MHH (No.: 12414-BO-S-2026). All study participants will be provided with detailed verbal and written information about the project objectives and data protection protocols before confirming their participation. Participation will only take place if the person expressly agrees to participate and signs or confirms a written or online declaration of consent. Additional written project information will be provided to participants in advance. All participants have the right to refuse, cancel, or withdraw their participation at any time before, during, or after the project without giving reasons and without incurring any consequences.

The data protection concept will include a detailed description of the data linkage procedure in WP1a. During the application preparation phase, AOKN and KKN exchanged preliminary variable lists to assess the data protection risk. The KKN’s trust area was involved in the application process and, if the application is approved, will consult with the relevant ministry to coordinate the procedure for the final data linkage. All parties involved will work closely together to address and minimize any remaining data protection concerns. The data collected as part of the project is subject to confidentiality, and all project staff are bound to data secrecy. The data collected from all work packages will be anonymized or pseudonymized in accordance with the applicable data protection guidelines, physically stored and/or stored on internal, secure servers at MHH, and used exclusively for this project. Data can be deleted at the request of participants, provided that this data has not (yet) been anonymized and can still be traced back to the individual.

## Discussion

### Expected results and transferability

The proposed ACTION project will generate relevant new and urgently needed scientific findings on the involvement of GPs in oncological aftercare. Particularly noteworthy is the scientific implementation of data linkage between KKN care data and AOKN billing data. Close cooperation between all parties involved has enabled the development of a low-threshold and data protection-compliant concept for data linkage, which is to be tested in the ACTION project. The following research projects can thus benefit directly from this, and the preparation and implementation of data linkage in healthcare research can be disseminated and promoted in order to answer relevant research questions in a targeted manner. The findings gathered in the project on experiences, perspectives, and factors that promote or hinder the involvement of GPs in oncological aftercare stimulate closer involvement of GPs. The resulting evidence- and consensus-based recommendations are transferable to the whole of Germany thanks to the involvement of experts from across the country. These findings can contribute to the development and, in the long term, the implementation of joint aftercare models, thereby improving oncological care in Germany in the long term. The long-term goal is to contribute to a nationwide improvement in oncological aftercare for patients, regardless of their individual access to cancer centers and specialists.

### Dissemination plans

The project results and recommendations will be presented at a digital closing symposium. Participants will include (a) the project team, (b) the cooperation partners and partner organizations, (c) experts in primary care and/or oncological care with a focus on aftercare, and (d) the interested public. In order to promote the accessibility and long-term preservation of research data and results at the scientific level, the project team will report comprehensively and transparently on the project and, regardless of the results achieved, will publish national and international publications, if possible, with open access. In addition, project results will be reported in publications relevant to the target group and at (inter)national congresses and conferences. Further opportunities for disseminating the results will be identified by involving experts from the field in the workshop and Delphi procedure in phase 2 of the project. In addition, the project results will be prepared in a manner appropriate to the target group and disseminated via the channels of the Institute of General Medicine and Palliative Medicine at MHH and, if applicable, the cooperation partners (including website, newsletter, and social media channels).

### Study risks

Recruiting GPs and oncological specialists can be difficult, depending on their attitude towards and experience with scientific projects and in view of the heavy time and personnel demands of their everyday care. It is possible that willingness to participate in the planned surveys in WP1b and 1c will be so low that the number of cases reached will be jeopardized. To compensate for this methodological challenge, the following measures are already planned at the time of application:

- Clear and precise preparation of the project material.
- Minimal time expenditure for participants (limiting the scope of the surveys to essential content).
- Multiple reminders and different methods with easy access (letter/fax with QR code, email with access link).
- Use of existing networks of the Institute of General Medicine and Palliative Medicine at MHH.
- Appeals via professional societies and associations (e.g., circular letter from HÄVN, publication in the Lower Saxony Medical Journal).

The case numbers specified in the application were calculated based on the topic and experience from previous projects involving the recruitment of these target groups and therefore represent a realistic target figure. Due to the high relevance of the topic and existing points of contact in everyday care, the project team assumes that interest in participation will be rather high. Based on previous project experience, the project team does not anticipate any major challenges in recruiting the target groups of oncological patients (WP1d) and experts (WP2a and WP2b). The participation of experts in phase 2 in the workshop (WP2a) and in the Delphi survey (WP2b) will be additionally supported by lump-sum allowances for loss of earnings and, if necessary, funds to reimburse travel expenses. In the implementation of WP1a, there is a risk in the practical implementation of data linkage. During the preparation of the application, detailed preparations and multiple consultations with the parties involved from KKN and AOKN took place in order to prepare and coordinate the procedure. Close cooperation with the KKN’s trust department further minimizes the risks in this regard, and there will be regular exchanges with working groups with practical experience in data linkage from the networks of the Institute of General Medicine and Palliative Medicine at MHH, KKN, and AOKN.

## Data Availability

No datasets were generated or analysed during the current study.

## Abbreviations

AOKN: Allgemeine Ortskrankenkasse Lower Saxony
CCC-N: Comprehensive Cancer Center Lower Saxony
GPs: general practitioners
KKN: Klinisches Krebsregister Niedersachsen (Lower Saxony Clinical Cancer Registry)
KVN: Kassenärztliche Vereinigung Niedersachsen (Association of Statutory Health Insurance Physicians of Lower Saxony)
MHH: Hannover Medical School
WP: working package
